# Serial analysis of 3D H-1 MRSI for patients with newly diagnosed GBM treated with combination therapy that includes bevacizumab

**DOI:** 10.1007/s11060-016-2229-3

**Published:** 2016-08-17

**Authors:** Sarah J. Nelson, Yan Li, Janine M. Lupo, Marram Olson, Jason C. Crane, Annette Molinaro, Ritu Roy, Jennifer Clarke, Nicholas Butowski, Michael Prados, Soonmee Cha, Susan M. Chang

**Affiliations:** 1 Department of Radiology and Biomedical Imaging, University of California, San Francisco, CA USA; 2 Department of Neurological Surgery, University of California, San Francisco, CA USA; 3 Helen Diller Family Comprehensive Cancer Center, University of California, San Francisco, CA USA; 4 Department of Neurology, University of California, San Francisco, CA USA; 5 Department of Bioengineering and Therapeutic Sciences, University of California, San Francisco, CA USA

**Keywords:** Metabolic imaging, MRSI, Newly diagnosed glioblastoma, Anti-angiogenic therapy, Survival

## Abstract

Interpretation of changes in the T1- and T2-weighted MR images from patients with newly diagnosed glioblastoma (GBM) treated with standard of care in conjunction with anti-angiogenic agents is complicated by pseudoprogression and pseudoresponse. The hypothesis being tested in this study was that 3D H-1 magnetic resonance spectroscopic imaging (MRSI) provides estimates of levels of choline, creatine, N-acetylaspartate (NAA), lactate and lipid that change in response to treatment and that metrics describing these characteristics are associated with survival. Thirty-one patients with newly diagnosed GBM and being treated with radiation therapy (RT), temozolomide, erlotinib and bevacizumab were recruited to receive serial MR scans that included 3-D lactate edited MRSI at baseline, mid-RT, post-RT and at specific follow-up time points. The data were processed to provide estimates of metrics representing changes in metabolite levels relative to normal appearing brain. Cox proportional hazards analysis was applied to examine the relationship of these parameters with progression free survival (PFS) and overall survival (OS). There were significant reductions in parameters that describe relative levels of choline to NAA and creatine, indicating that the treatment caused a decrease in tumor cellularity. Changes in the levels of lactate and lipid relative to the NAA from contralateral brain were consistent with vascular normalization. Metabolic parameters from the first serial follow-up scan were associated with PFS and OS, when accounting for age and extent of resection. Integrating metabolic parameters into the assessment of patients with newly diagnosed GBM receiving therapies that include anti-angiogenic agents may be helpful for tracking changes in tumor burden, resolving ambiguities in anatomic images caused by non-specific treatment effects and for predicting outcome.

## Introduction

The current standard of care for patients with newly diagnosed glioblastoma (GBM) is maximal safe resection, followed by radiation therapy and temozolomide [1]. A variety of investigational agents are being considered in conjunction with this strategy in order to improve outcome. Evaluation of combination therapies is complicated by differences in their mechanisms of action that lead to difficulties in assessing the spatial extent of tumor and ambiguities in the interpretation of conventional anatomic images. One such effect that has been reported to occur in up to 30 % of patients treated with radiation and temozolomide is pseudoprogression, which is characterized by an increase in the size of the enhancing lesion that subsequently disappears without further intervention [2].

Given that GBM are characterized by abnormal vasculature, there has been great interest in using treatment regimens that include anti-angiogenic agents [3–5]. The dramatic reduction in the size of the contrast enhancing (CE) lesion that was observed following treatment of recurrent GBM with bevacizumab was very encouraging and led to conditional approval for its use. Subsequent studies in populations of patients with newly diagnosed GBM have shown longer progression free survival but have not established a significant improvement in overall survival [6, 7]. For cases where the treatment regime includes such anti-angiogenic agents, it is not clear whether early reductions in the CE lesion are indicative of successful treatment or if they are associated with pseudo- or true response.

The ambiguities in interpreting changes in the size of the CE lesion have highlighted the need to develop alternative approaches for assessing treatment response. The RANO criteria are a first step in that direction [8], as they consider changes in the cross sectional diameter of both the CE and T2 lesion. A further suggestion has been that estimating the three dimensional (3D) volumes of the anatomic would provide a more precise assessment of their absolute and relative changes with time. While providing more precise quantification of lesion size is definitely of interest, it is not clear whether this would be able to address pseudoprogression and pseudoresponse. Having an imaging modality that makes it possible to track biological response to therapy is likely to be a more promising approach. Previous studies that applied H-1 MR spectroscopic imaging (MRSI) to evaluate patients with GBM have shown that the magnitude and spatial extent of regions with abnormal metabolism is often quite different from areas corresponding to the CE and T2 lesions [9–12]. This suggests that metabolic imaging can provide complementary information about changes in tumor burden that would be helpful in assessing response to therapy.

The purpose of this study was to obtain 3D H-1 MRSI data from a cohort of patients with newly diagnosed GBM being treated with radiation therapy, temozolomide, erlotinib and bevacizumab in order to evaluate treatment induced changes in the metabolic characteristics of the lesion. The hypothesis being tested was that levels of choline, creatine, *N*-acetylaspartate (NAA), lactate and lipid change in response to treatment and that metrics describing these characteristics are associated with survival.

## Methods

Thirty-one patients with pathologically confirmed newly diagnosed GBM, who were being treated as part of a single institution Phase II clinical trial [13] were recruited for this study. External beam radiation therapy (RT) was initiated within 5 weeks of diagnosis and delivered an average dose of 60 Gy to the tumor site in 2-Gy fractions over a 6-week period. Temozolomide was given concurrently and after RT at a daily dose of 75 mg/m^2^. Patients who were not on anti-epileptic drugs received 150 mg/day of erlotinib and patients who were on anti-epileptic drugs received 500 mg/day starting on day 1 of RT. Bevacizumab was prescribed at a dose of 10 mg/kg IV every 14 days starting in week 2 of RT. All patients participating in the study gave informed consent, according to the guidelines of our institutional review board. Overall survival (OS) was calculated as the time to death from the start of treatment and progression free survival (PFS) as the time from the start of treatment to the examination at which the patient was determined to have progressed based upon the RANO criteria or the time of death if there had been no prior progression [8].

### Imaging protocol

Patients received MR exams at multiple time points, including following surgical resection but prior to RT (pre-RT), between 3 and 5 weeks into treatment (mid-RT), within 2 weeks after completion of RT (post-RT), and at the next follow-up scan that was approximately 4 months after the baseline time point (Fup1). All scans were obtained with a 3 T MR scanner (GE Healthcare, Milwaukee, WI), the body coil for transmission and an 8-channel phased array coil for reception. Standard anatomical imaging comprised axial T2-weighted fluid attenuated inversion recovery (FLAIR) images (TR/TE/TI = 9500/122/2375 ms, matrix = 256 × 256, slice thickness = 3 mm, FOV = 24 × 24 cm), and pre- and post-contrast volumetric T1-weighted inversion recovery spoiled gradient echo images (TR/TE = 8.86/2.50 ms, matrix = 256 × 256, slice thickness = 1.5 mm, FOV = 24 × 24 cm, TI = 400 ms, Flip angle = 15°).

Lactate edited 3D H-1 MRSI data [14] were obtained with CHESS water suppression, very selective saturation (VSS) outer volume suppression and PRESS volume selection (TR = 1104 or 1300 ms, TE = 144 ms, acquisition matrix of 16 × 16 × 16 or 18 × 18 × 16, nominal spatial resolution of 1 cm^3^). Phase encoding was applied in the right-left and anterior-posterior directions with fly-back trajectories in the superior-inferior direction. An over-PRESS factor of 1.5 was applied with VSS bands to avoid chemical shift artifacts and to suppress residual lipid signals. The total acquisition time was 9.5 min, with 712 complex spectral points read indirectly using the EPI train of gradient pulses and spectral bandwidth of 988 Hz. The PRESS selected volume was defined using the anatomic images and generally covered the lesion and 200–500 cc of normal tissue. Areas with sharply varying magnetic susceptibility and lipid contamination were avoided whenever possible.

### Processing MRI and MRSI data

After each examination, the DICOM images and raw spectral data were transferred offline for post-processing. The volumetric post-contrast T1 weighted image was used as a reference and the other images were aligned to the same coordinate system through rigid body transformations that maximized the normalized mutual information [2, 15]. The CE lesion was manually defined on the post-contrast T1 weighted images. Any enhancement that was also present on the pre-contrast T1 images was assumed to represent acute blood products and was excluded. The T2 lesion was segmented based on the region of hyperintensity on FLAIR images. The resection cavity was excluded from all ROIs. T1 subtraction images [16] were generated from the pre- and post-contrast T1-weighted images and the T1s lesion was identified by thresholding the region of hyperintensity within the T2 region. Normal appearing brain was identified by brain extraction, followed by removal of regions within the T2 lesion ROI.

**Fig. 1:**
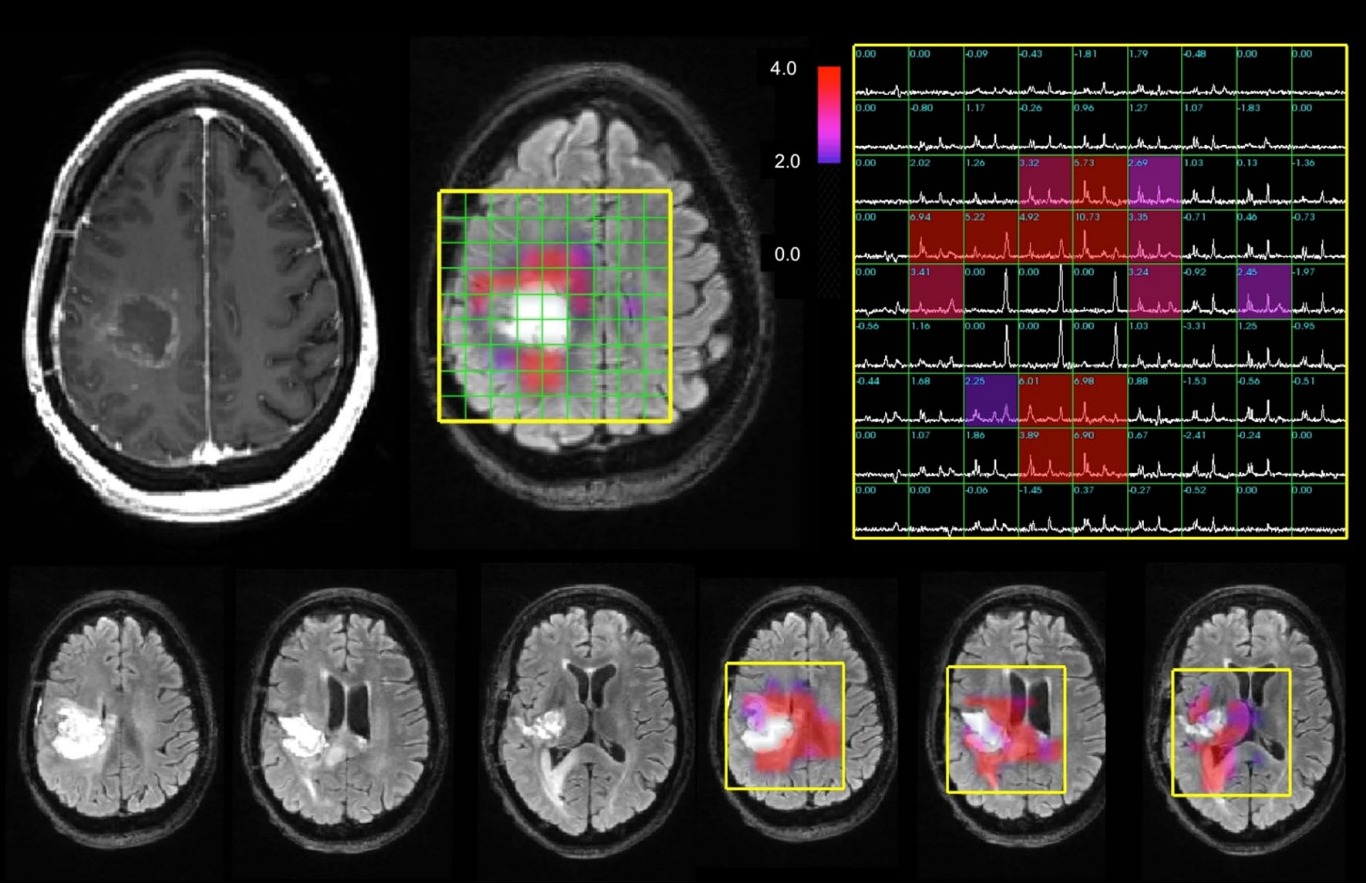
Anatomic images, spectra and CNI color overlays from the Fup1 scan for a 47 year old female patient with a KPS of 90, sub-total resection, a low PFS of 159 days and a low OS of 206 days. The CNI maps were thresholded to show only values that were 2 or higher in order to highlight the metabolic lesion. The median and maximum CNI were 4.8 and 19.9. Note the high lipid peaks in the cavity, which are surrounded by areas with increased choline and decreased NAA that corresponded to the metabolic lesion. The residual CE volume was 3 cm^3^ is minimal and corresponds mainly to a thin rim around the resection. The volume of the T2 lesion at this time point is 29 cm^3^. As is shown from the FLAIR images and CNI overlays from the three lower slices, the metabolic lesion is relatively large and gives a clear picture of regions that are likely to reflect recurrent/residual tumor

The spectral data were processed as described previously [17, 18] to provide levels of choline, creatine, NAA, lactate and lipid. The choline-NAA index (CNI) is a metric developed to describe the deviation of choline and NAA in regions with normal metabolite levels in the same individual [19, 20]. The calculation is performed using an automated, iterative procedure that uses choline and NAA levels in the spectral array from each examination separately and is independent of the anatomic images. Once the CNI map had been generated for each patient and time point, their metabolic lesion was defined as the region having values greater than or equal to 2 (CNI2). Figure 1 shows an example of the spectral data and CNI overlays from the Fup1 scan for a patient with a relatively large metabolic lesion at this time point. The CNI intensities were interpolated to the resolution of the anatomic images for display purposes and the overlay was thresholded to show only values above 2 in order to highlight the spatial extent of the metabolic lesion. The choline to creatine index (CCrI) was estimated in a similar manner, but with the definition of abnormal voxels being based upon those that had been identified during the CNI calculation.

To be able to compare across serial exams, levels of choline, creatine, and NAA intensities were normalized by their median value in voxels from normal appearing brain (nCho, nCr and nNAA). Signals from lipid and lactate peaks were normalized with the median NAA from voxels in normal appearing brain (nLac and nLip). Parameters considered for analysis were the volume of the CNI2 and the median, maximum and summation of voxel values for the CNI, CCrI, nLac and nLip from within the CNI2 regions at each of the four time points.

### Statistical analysis

Serial changes in imaging parameters were evaluated using Wilcoxon signed rank sign tests for paired analysis. Association with PFS and OS was assessed using Cox proportional hazards analysis, taking into account patient age and extent of resection. The cut-off for defining a significant result in this exploratory study was a P value of 0.05.

## Results

The median PFS for these patients was 404 days with 27 events and 4 subjects censored. The median OS was 603 days with 23 events and 8 subjects censored. There were 16 females and 15 males: median age was 52 years with a range of 21 to 75, median KPS was 90 with a range of 60–90. There were 9 patients who were designated based upon clinical criteria as having received a gross total resection, 17 who had a sub-total resection and 5 who were characterized as having a biopsy.

### Changes in anatomic lesion volumes

**Fig. 2:**
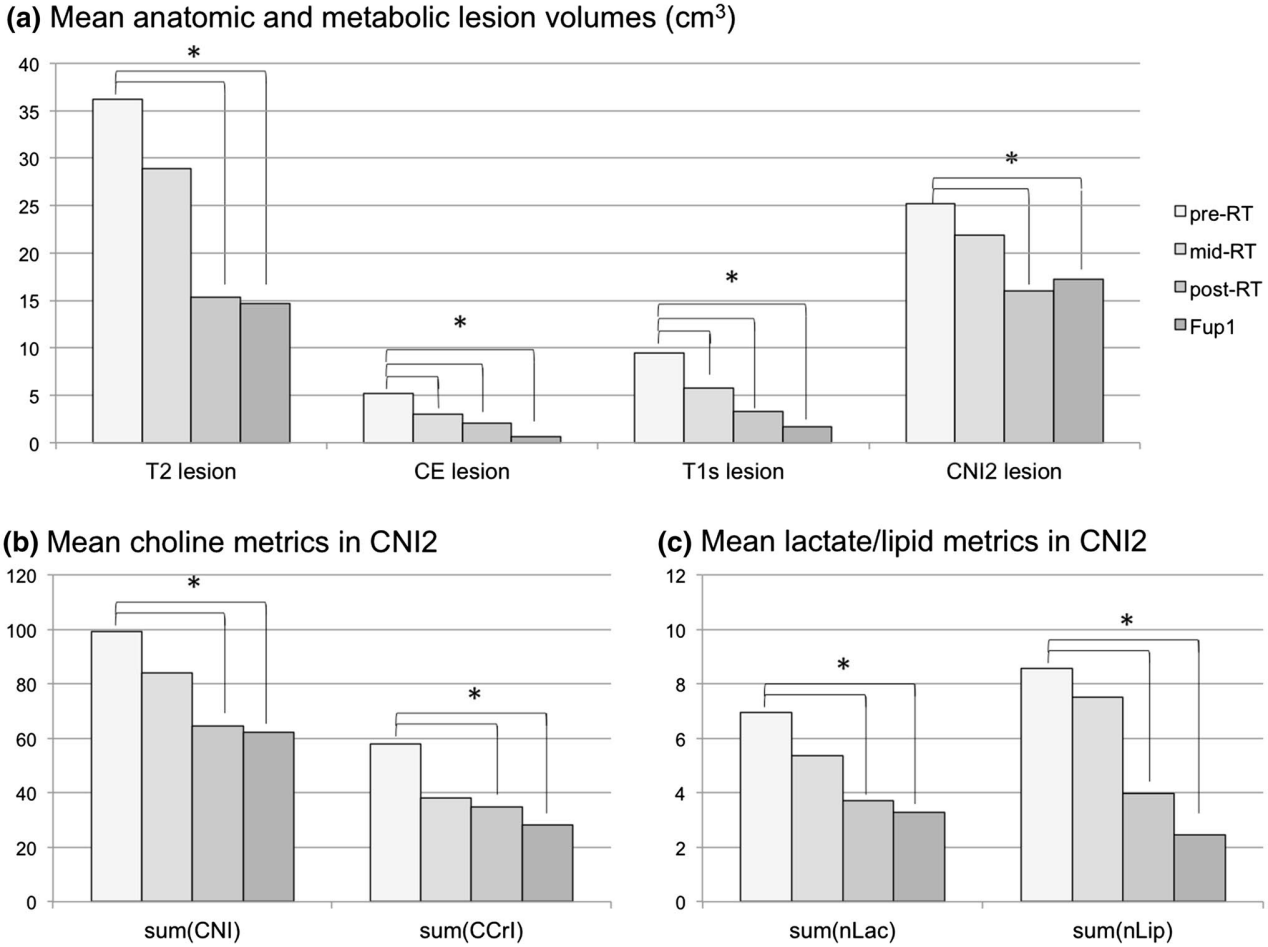
Changes in mean anatomic, choline, lactate and lipid parameters for patients with scans at all four time points (pre-, mid-, post-RT and Fup1). The *asterisks* denote time points at which the change from pre-RT in the relevant parameter was statistically significant. The sum values are integrals of the various parameters from voxels within the region with CNI >2. There were 21 subjects with anatomic data and 19 subjects with metabolic data at all 4 time points

For the 22 patients who had anatomic scans at all 4 time points there were overall reductions in the volumes of the anatomic lesions with treatment. The mean pre-RT T2 lesion volume was 36.2 cm^3^, which subsequently decreased to 28.9 cm^3^ at mid-RT (p > 0.05), to 15.3 cm^3^ at post-RT (p < 0.0001) and to 14.7 cm^3^ at Fup1 (p = 0.0002). The mean CE lesion volume decreased from 5.2 cm^3^ at pre-RT to 3.0 cm^3^ at mid-RT (p = 0.018), 2.1 cm^3^ at post-RT (p = 0.0002) and 0.7 cm^3^ at Fup1 (p < 0.0001). The mean T1s volume was obtained by thresholding the T1 subtraction image and showed similar changes to the CE lesion volumes but was overall larger in size at all four time points (mean volumes of 9.4, 5.8, 3.3 and 1.7 cm^3^ respectively). These results are represented in the bar graphs in Fig. 2.

### Changes in metabolic parameters

At pre-RT, the CNI2 lesion volumes were much larger than the CE and T1s lesions (mean of 25.2 vs. 5.2 and 9.1 cm^3^ respectively). Although many of the T2 lesion volumes at pre-RT were larger than the CNI2 lesions, the mean overlap was only 11.2 cm^3^. This indicates that there were sub-regions of FLAIR hyperintensity with CNI <2, as well as regions of normal appearing brain with elevated CNI. For the 19 patients with metabolic imaging data at all 4 scans, the changes in mean CNI2 volume from pre-RT to post-RT and from pre-RT to Fup1 were significant (from 25.2 to 16 cm^3^, p = 0.0017 and 17.2 cm^3^, p = 0.0145 respectively). The differences in spatial extent of the anatomic and metabolic lesions became even more extreme after treatment, with the overlap between the CNI2 and T2 lesion volumes being 4.6 cm^3^ at post-RT and 3.8 cm^3^ at Fup1. As can be seen from the lower bar graphs in Fig. 2, the sum(CNI), sum(CCrI), sum(nLac) and sum(nLip), which represent how abnormal the metabolite levels were within the CNI2 region from each time point relative to values in normal brain showed a similar pattern of changes.

### Serial results from individual patients

**Fig. 3:**
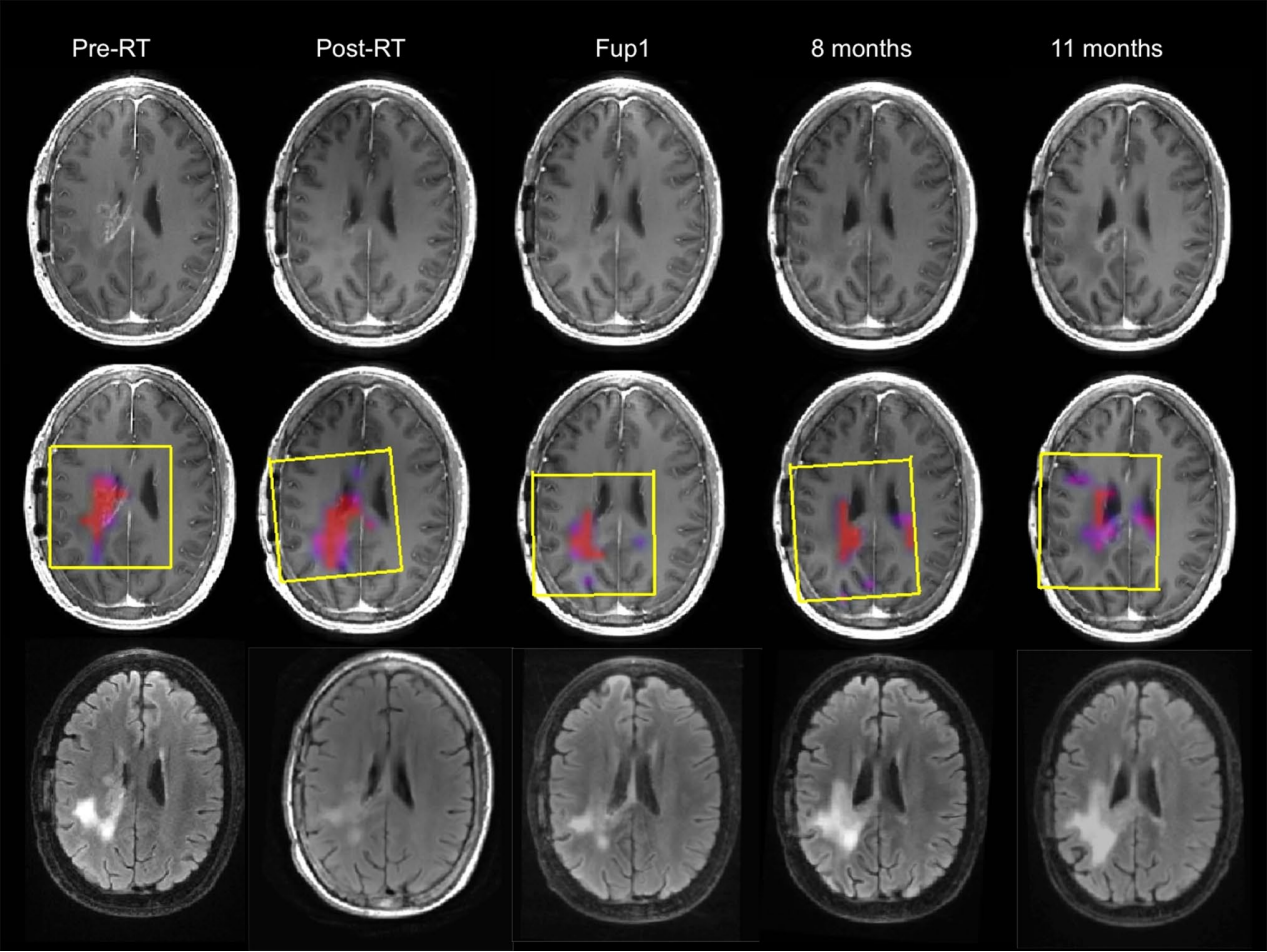
Serial post-Gad T1-weighted, FLAIR images and overlaid CNI maps for a male patient who was 47 years old, had a PFS of 330 days and OS of 706 days. The *yellow boxes* indicating PRESS selected volumes are oblique for follow-up scans because the 3D imaging and spectral data were post-processed to register them to the pre-RT exam in order to aid in making visual comparisons

Figure 3 shows an example of the serial anatomic images and overlays of CNI maps from pre-RT, post-RT, Fup1 and at two later time points prior to progression for a 47 year old patient who had received a sub-total resection, and had a KPS of 70, with PFS of 330 days and OS of 706 days. The images on the upper panel are from the post-contrast T1-weighted sequence and show that the enhancing lesion virtually disappears by post-RT (from an overall volume of 6.7 cm^3^ at pre-RT to 0.5 cm^3^ at Fup1) and a small region of new enhancement appears in the corpus callosum at the 11month follow-up scan. The FLAIR images on the bottom panel show a large T2 lesion at pre-RT (overall volume of 55 cm^3^) with two components, one being brighter and the other being less intense. The T2 lesion was smaller at the post-RT and Fup1 scan but was beginning to impinge upon the corpus callosum at the 8 month scan and then continued expanding in the 11 month scan. The CNI2 region represented by the color overlay images in the middle panels included only a portion of the T2 lesion. The maximum CNI at pre-RT was 20.5 and at Fup1 it was 18.6, suggesting that, despite the lack of enhancement, there was substantial residual tumor. On the 8 month and 11 month scans the CNI2 lesion increased in size and had clearly crossed the corpus callosum, but was still smaller than the overall T2 lesion.

**Fig. 4:**
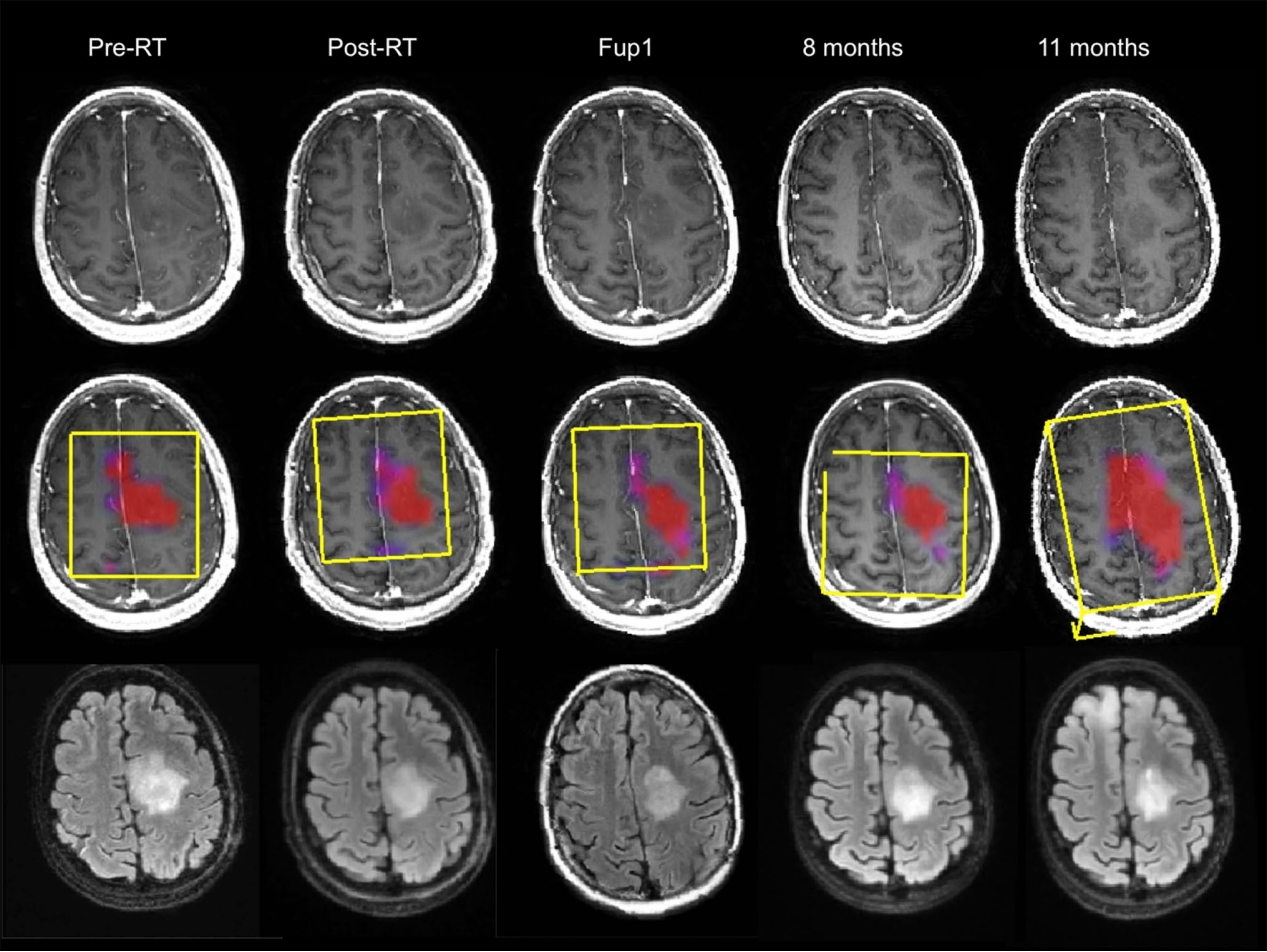
Serial post-Gad T1-weighted, FLAIR images and overlaid CNI maps for a female patients who was 38 years old, had a PFS = of 360 days and OS of 434 days. The yellow boxes indicating PRESS selected volumes are oblique for follow-up scans because the 3D imaging and spectral data were post-processed to register them to the pre-RT exam in order to aid in making visual comparisons

Figure 4 shows a similar time course for a second patient who was 38 years old, had a KPS of 90, a biopsy only, similar PFS of 360 days and a shorter OS of 434 days. The overall CE lesion volume at the pre-RT scan was 5.5 cm^3^ but reduced to <1 cm^3^ on subsequent scans. The T2 lesion had an overall volume of 37 cm^3^ at pre-RT, reducing at the post-RT scan and 4 month scans to 25 cm^3^ and 20 cm^3^ respectively. At the 11 month scan, the T2 lesion volume was similar in size on the axial slice shown but had expanded on inferior slices. The metabolic lesion had high CNI values at all time points (the maximum CNI at pre-RT was 25.6 and at Fup1 was 22.9). At the initial time points, the region with CNI >2 covered the majority of the T2 lesion, suggesting that there was substantial non-enhancing tumor. While the CNI2 lesion changed in shape during the initial 8 months, it was fairly stable in size until the 11month scan when it became substantially larger and extended beyond the T2 lesion. There was no evidence of enhancement on the post-Gadolinium T1-weighted at this time. Once more, the metabolic data were helpful in providing confidence that the increase in the volume of the T2 lesion corresponded to infiltrative tumor. The large increase in the CNI2 lesion volume at progression was consistent with the relatively short OS.

### Association with survival

The only anatomic imaging parameter that showed an association with outcome was the volume of the CEL at post-RT for PFS (p = 0.029). In contrast, a large number of the metabolic imaging parameters considered in the analysis were associated with PFS and OS at the Fup1 (4 month) scan (see Table 1). If the population was divided into cohorts with survival either shorter or longer than the median value, it was observed that there were significant reductions in metabolite levels for both cohorts and that these changes were observed at both the post-RT and the Fup1 scans. This was true when using both the median PFS and median OS to define the cut-points.

**Table 1:** Association of imaging and metabolic parameters with survival based upon Cox proportional hazards analysis, adjusting for age and extent of resection

Parameters	Progression free survival				Overall survival			
	Pre-RT	Mid-RT	Post-RT	Fup1	Pre-RT	Mid-RT	Post-RT	Fup1
Anatomic imaging								
Volume (T2)	NS	NS	NS	NS	NS	NS	NS	NS
Volume (CE)	NS	NS	0.0299	NS	NS	NS	NS	NS
Volume (T1s)	NS	NS	NS	NS	NS	NS	NS	NS
Metabolic imaging								
Volume(CNI2)	NS	NS	NS	0.0046	NS	NS	NS	0.0080
Sum(CNI)	NS	NS	NS	0.0034	NS	NS	NS	0.0033
Sum(CCrI)	NS	NS	NS	0.0033	NS	NS	NS	0.0040
Sum(nLac)	NS	NS	NS	0.0019	NS	NS	NS	0.0010
Sum(nLip)	NS	NS	NS	0.0001	NS	NS	NS	0.0034

Metrics with p < 0.05 were considered to be significant but all of the metabolic parameters have much smaller P values. The number of subjects with anatomic imaging data at the each of the time points was 29, 25, 25 and 27, while the number of subjects with metabolic imaging data was 28, 24, 21 and 26. There were 4 subjects censored for PFS and 10 subjects censored for OS

## Discussion

The results of our study highlighted the substantial changes in anatomic lesion volumes that are seen in treatments that include anti-angiogenic agents such as bevacizumab. The RANO criteria for assessing response to therapy in patients with GBM depend upon changes in cross sectional diameter of the CE lesion, with secondary evaluation of the T2 lesion [8]. This is problematic for treatments including anti-angiogenic agents because the size of the enhancing portion becomes too small for further evaluation, while the T2 lesion may include a mixture of non-enhancing tumor and non-specific treatment effects. Of interest in the current study is that the major reduction in the T2 lesion volume occurred between the mid- and post-RT scan, whereas the volumes of the CE and T1 subtraction lesions were significantly reduced at the mid-RT scan and continued decreasing for the next two scans. These observations are consistent with prior reports of changes in lesion volumes that are observed following treatment with anti-angiogenic agents and have been attributed to normalization of the vasculature in the tumor and associated reduction in edema [2–7]. Of interest is that the only anatomic parameter associated with PFS was the volume of the enhancing lesion at the post-RT scan. It is presumably a reflection of whether the lesion was sensitive and showed an immediate response to this agent.

An alternative approach that has been suggested for assessing changes in tumor burden is to use 3-D H-1 MRSI to evaluate regions with abnormal metabolism [22–24]. The CNI and related metrics such as the ratio of choline to NAA have previously been shown to be increased in tumor [20], and to detect areas of infiltrative tumor outside of the CE lesion [21]. This may be important for targeting radiation and other focal therapies, as well as monitoring response to treatment [22–29]. Of particular interest is our prior study of patients with newly diagnosed GBM being treated with radiation and temozolomide [12], which indicated that metabolic parameters reduced from pre- to post-RT and that values obtained from the post-RT scan were associated with overall survival.

The results of the current study indicate that there is only partial overlap between the CNI2 and T2 lesions, with there being regions of FLAIR hyperintensity that are not metabolically abnormal and regions of normal appearing brain with elevated CNI values. This suggests that the spatial information provided by the metabolic data is complementary to both types of anatomic images. The color overlays of the CNI maps that are presented in the figures are helpful in relating the metabolic and anatomic data in a visual manner. A critical factor to note here is that although 9/31 patients in our study were assessed based on clinical criteria as having a gross total resection, all of the subjects had regions with metabolic parameters that were consistent with tumor.

Another property of the MRSI data is that they provide measurements of levels of multiple metabolites that can be quantified and tracked with time. The reductions in parameters reflecting abnormal levels of choline relative to NAA (CNI) and creatine (CCrI) suggest that the tumor cellularity is decreasing and is therefore responding to therapy. Prior studies that used MRSI to study patients being treated with cediranib [30], radiation and temozolomide [12] and other treatment strategies [13, 31, 32] have also showed a reduction in choline related parameters. The lower levels of lipid and lactate (nLip and nLac) within the metabolic lesion at the post-RT and the next follow-up scans indicate that the tumor is becoming better oxygenated, which is consistent with the impact of vascular normalization [21]. The associations of metabolic parameters from the follow-up scan with PFS and OS are encouraging in terms of providing surrogate measures of the effectiveness of different treatments. Including consideration of these metabolic parameters into the RANO criteria would be especially helpful for circumstances where the T2 lesion has increased in size and it is uncertain whether this is due to tumor progression. While the spatial resolution of MRSI is currently limited by signal to noise and acquisition times to approximately 1 cm^3^, the impressive results that have been obtained in this study suggest that it provides complementary information to other imaging modalities. Future clinical studies should be designed to include the acquisition of metabolic imaging data in order to determine whether these results hold for a larger cohort of patients.

## Conclusions

This study supports the hypothesis that levels of choline, creatine, NAA, lactate and lipid change in response to treatment and that metrics describing these characteristics are associated with survival. Integrating 3D H-1 MRSI into the serial assessment of patients with GBM who are receiving therapies that include anti-angiogenic agents may therefore be helpful for resolving ambiguities caused by non-specific treatment effects and for predicting outcome.
